# Rare Synchronous Occurrence of Maxillary Glandular Odontogenic Cyst and Low-Grade Mucoepidermoid Carcinoma in a Young Adult: A Case Study

**DOI:** 10.7759/cureus.111389

**Published:** 2026-06-23

**Authors:** Asish K Das, Subhasish Burman, Pranay G Tumsare, Abhijit Maji, Inam Uddin

**Affiliations:** 1 Oral and Maxillofacial Surgery, Dr. R. Ahmed Dental College and Hospital, Kolkata, IND

**Keywords:** glandular odontogenic cyst (goc), low-grade mucoepidermoid carcinoma, mucoepidermoid carcinoma (mec), odontogenic cyst, salivary gland tumor, synchronous occurence, young adult

## Abstract

A glandular odontogenic cyst (GOC) is an infrequent developmental cyst originating from odontogenic tissues, whereas low-grade mucoepidermoid carcinoma (LGMEC) is an uncommon malignant epithelial tumor of the minor salivary glands. The simultaneous presence of these two conditions in adjacent maxillary sites is extremely rare and presents significant challenges in both diagnosis and treatment. This case study reports on a 23-year-old female patient who exhibited swelling involving the area from the second premolar through the second molar region on the right side of the maxilla, affecting both buccal and palatal areas. Clinical and radiographic assessments indicated a buccal lesion, likely cystic in nature, and a distinct palatal mass, possibly of salivary gland origin. Histopathological analysis confirmed the presence of a GOC on the buccal side and an LGMEC on the palatal side. The management approach was tailored according to the unique biological characteristics of each lesion. The GOC was managed through enucleation, while the LGMEC required extensive local excision to ensure clear margins. Both procedures were successfully performed under local anesthesia, resulting in uneventful healing and no signs of recurrence over a 12-month follow-up period. This case underscores the critical need for comprehensive evaluation of maxillary swellings, awareness of rare synchronous pathologies, and the necessity for tailored surgical strategies based on definitive histopathological diagnoses. Early detection and appropriate treatment can significantly enhance patient outcomes in such unusual cases.

## Introduction

Glandular odontogenic cyst (GOC) is an uncommon developmental odontogenic cyst. It was initially reported by Padayachee and Van Wyk in 1987 under the designation ‘sialo-odontogenic cyst’ [1]. In 1988, Gardner proposed the term ‘glandular odontogenic cyst,’ and the World Health Organization subsequently classified it as a separate pathological entity in 1992 [2]. This cyst is characterized by local aggressiveness, unilocular or multilocular radiolucency, and a histological pattern featuring mucous cells, duct-like spaces, and epithelial whorls.

Mucoepidermoid carcinoma (MEC) was first documented by Stewart et al. in 1945 [3]. MEC represents approximately 5-10% of all salivary gland cancers. It most commonly occurs in the parotid gland, accounting for 89.6% of cases, while involvement of the submandibular and sublingual glands is less common, occurring in 8.4% and 0.4% of cases, respectively [4]. Approximately one-third of MECs develop in minor salivary glands, with the palate being the most common site. Histologically, MECs are classified into low-grade (48%), intermediate-grade (13.3%), and high-grade (38.7%) categories [5]. LGMEC arising in the minor salivary glands of the palate is an uncommon condition, typically presenting as a painless, slow-growing mass.

Given the overlapping features of GOC and LGMEC, accurate diagnosis often necessitates careful histopathological correlation. Reports of simultaneous or closely situated occurrences of GOC and LGMEC are exceedingly rare, with only isolated cases noted in existing literature [6,7]. Their co-occurrence presents diagnostic challenges due to the similar mucous and glandular components present in both lesions.

This case study highlights a unique presentation of GOC and LGMEC in close anatomic proximity in the right posterior maxilla. It discusses the clinical, radiological, and histopathological features, as well as the surgical management, and briefly reviews the available literature on these uncommon pathologies.

## Case presentation

A 23-year-old female patient presented with a six-month history of swelling in the right posterior palatal region (Figure 1).

**Figure 1:**
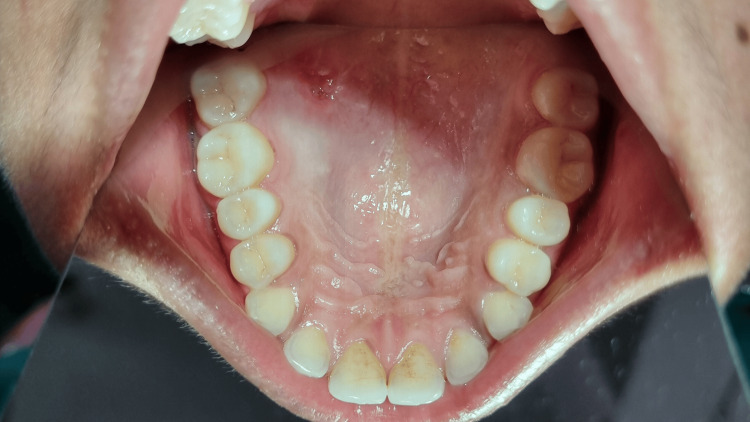
Pre-operative clinical presentation

The swelling measured approximately 2 cm x 3 cm, firm, non-tender, non-ulcerated, and non-fluctuant, and it was extending from the second premolar to the second molar region. It increased in size over the first three months and then stabilized. There was no bleeding or discharge from the swelling.

Cone beam computed tomography (CBCT) revealed a soft tissue mass in the right posterior palatal region without palatal bone involvement, alongside a well-defined radiolucency in the right posterior maxillary alveolar region, extending from 14 to 17 (Figure 2).

**Figure 2:**
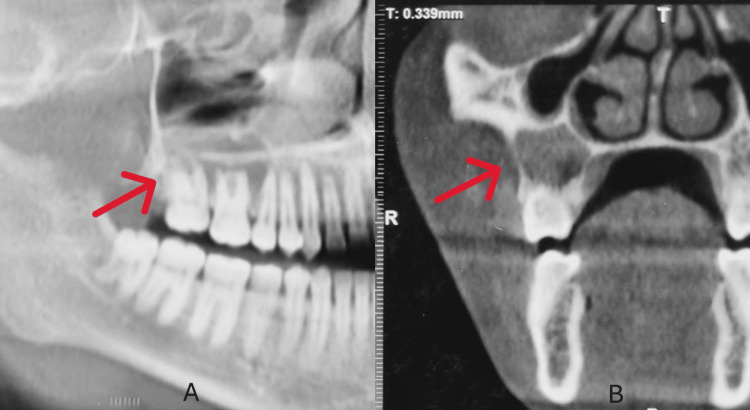
Pre-operative cone beam computed tomography (CBCT) scan A) Orthopantomogram view with arrow showing well defined radiolucency; B) Coronal section with arrow showing radiolucency within right posterior maxillary alveolar region.

Aspiration of the lesion in the right posterior maxilla yielded mucinous, straw-colored fluid. In contrast, the palatal mass aspiration was negative, suggesting a cystic lesion in the maxillary alveolus and a tumorous mass in the palatal soft tissue. The teeth associated with these lesions were vital.

Incisional biopsies were performed on both lesions. Histopathological evaluation of the maxillary alveolar cystic lesion revealed mucous cells, ciliated cells, duct-like structures, and epithelial spheres characteristic of a GOC. The palatal mass showed mucous, epidermoid, and intermediate cells arranged in both cystic and solid patterns, confirming LGMEC (Figure 3).

**Figure 3:**
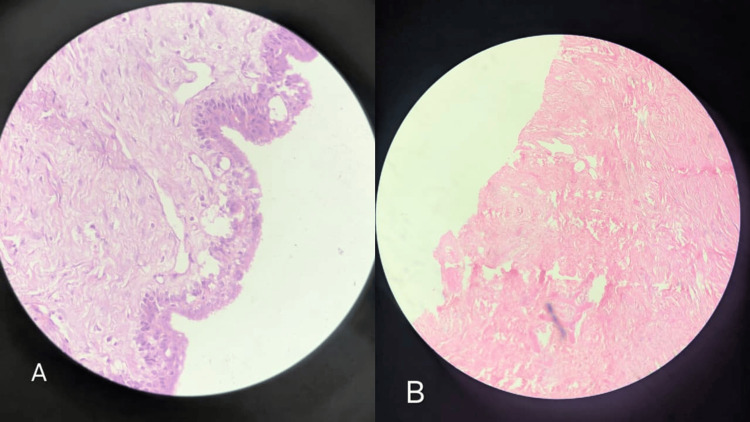
Histopathological images Suggestive of glandular odontogeic cyst (GOC) of the tissue taken from alveolar aspect of the lesion (A), and low-grade mucoepidermoid carcinoma (LGMEC) of the tissue taken from palatal aspect of the lesion (B).

Under local anesthesia, the GOC was completely enucleated after elevating a full-thickness mucoperiosteal flap via a crevicular incision from teeth 14 to 17, with an anterior release mesial to 14 (Figure 4).

**Figure 4:**
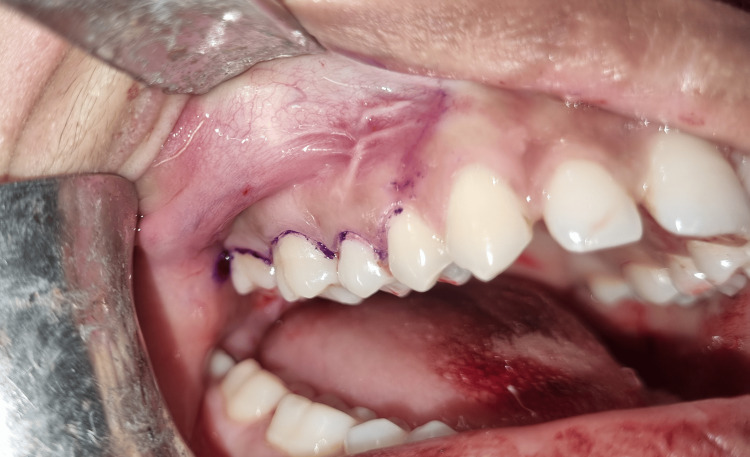
Incision marking on the buccal aspect

The cystic lining was thick and firm (Figure 5).

**Figure 5:**
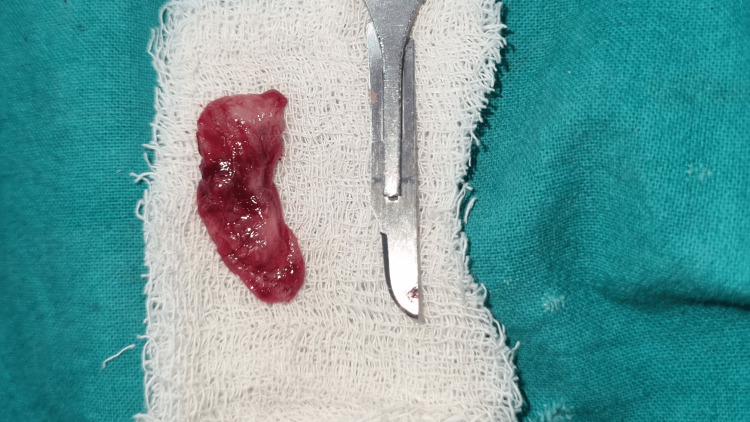
Enucleated cystic lesion

Teeth 15, 16, and 17 were extracted. The palatal LGMEC underwent wide local excision starting from the distal of tooth 14 anteriorly, through the mid-palatal raphe medially, and from the junction of the hard and soft palate posteriorly, ensuring adequate safety margins (Figure 6).

**Figure 6:**
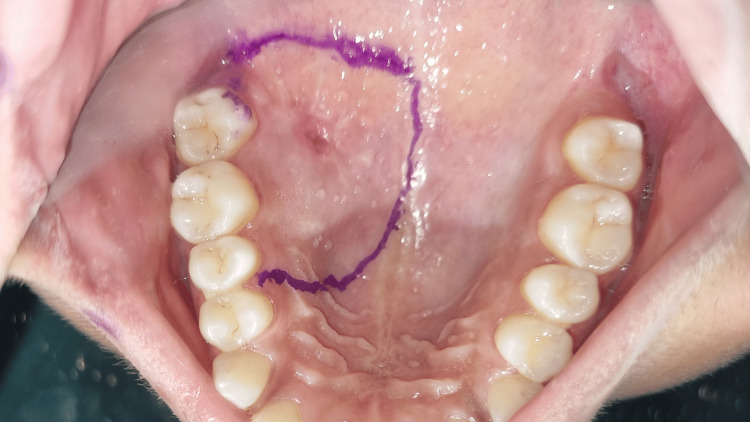
Incision marking on the palatal aspect

During the procedure, the greater palatine artery was ligated to manage mild bleeding. The excised specimen was firm and resilient (Figure 7).

**Figure 7:**
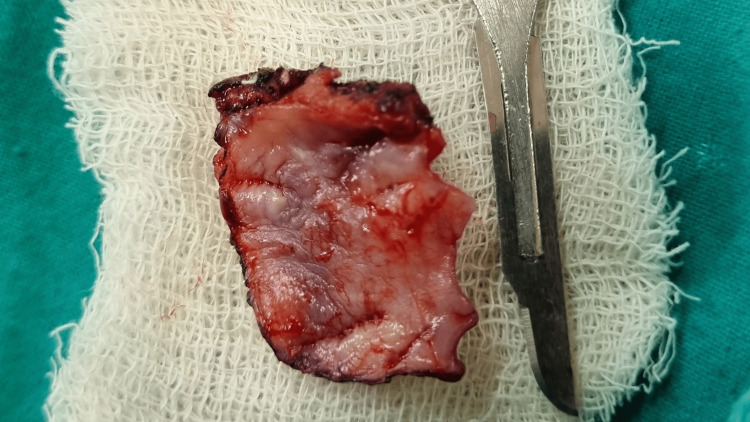
Excised specimen on the palatal aspect

No bony erosion was observed post-excision (Figure 8).

**Figure 8:**
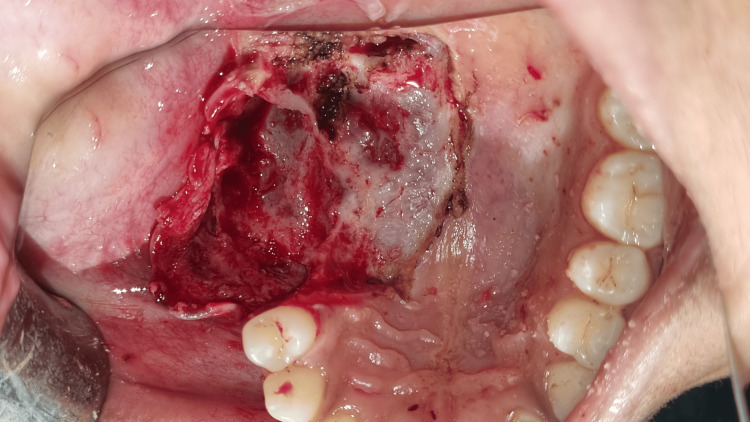
Surgical site post excision

A peripheral osteotomy followed by chemical cauterization with modified Carnoy’s solution was performed (Figure 9).

**Figure 9:**
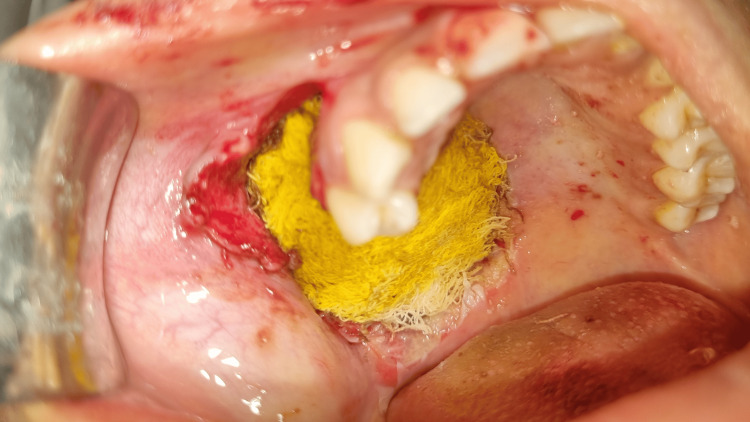
Chemical cauterization using modified carnoy's solution

A bismuth iodoform paraffin paste (BIPP)-impregnated gauze pack was sutured with simple interrupted sutures for secondary healing (Figure 10), and a pre-operatively fabricated cover plate was applied (Figure 11).

**Figure 10:**
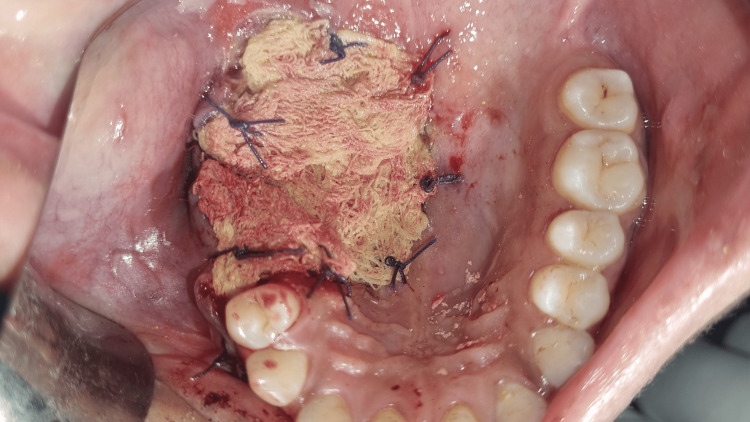
BIPP impregnated gauze pack sutured within the surgical defect BIPP: bismuth iodoform paraffin paste.

**Figure 11:**
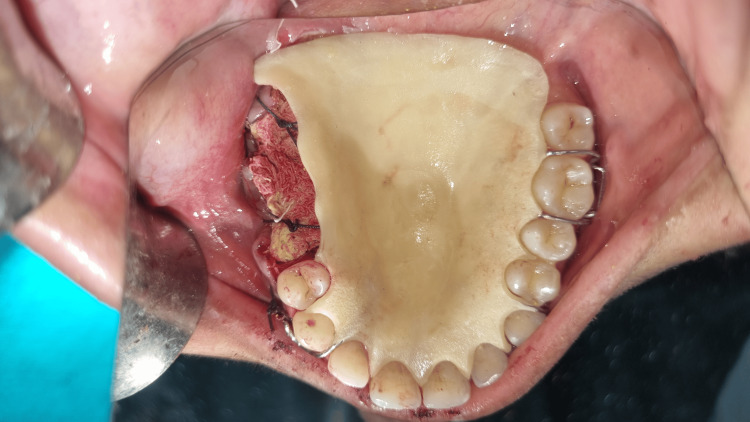
Prefabricated cover plate placement

Healing was uneventful (Figure 12).

**Figure 12:**
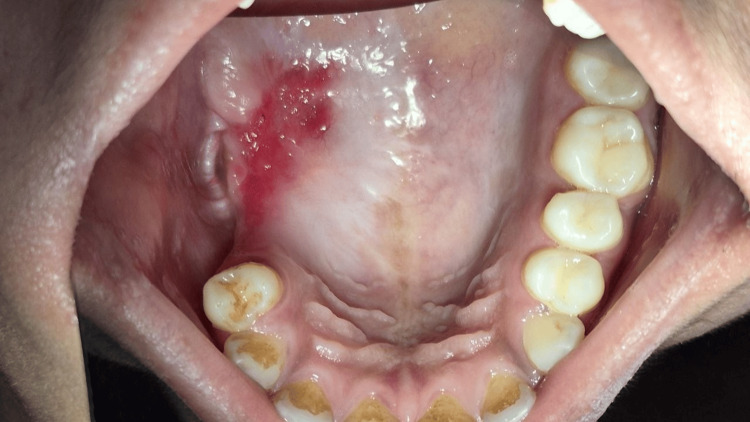
Post-operative clinical presentation

The patient remained disease-free throughout a stringent 12-month follow-up period (Figure 13).

**Figure 13:**
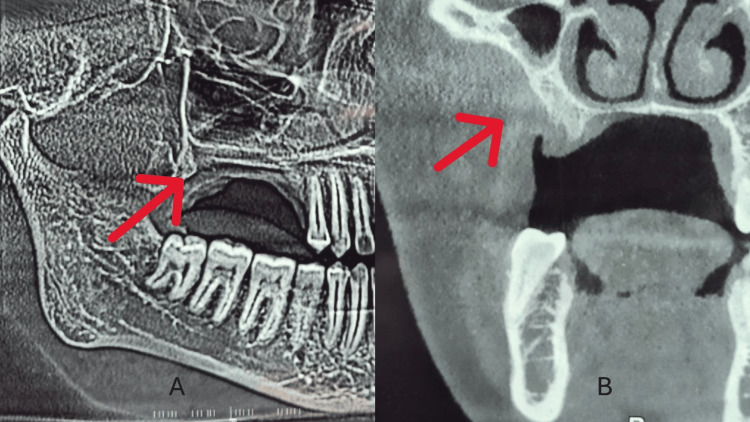
Post-operative cone beam computed tomography (CBCT) scan A) Orthopantomogram view with arrow showing post-operative surgical site; B) Coronal section with arrow showing surgical site without any breach in maxillary sinus and nasal cavity.

## Discussion

GOC is an uncommon lesion that accounts for only 0.012-1.3% of all cysts affecting the facial skeleton [7]. GOC is most commonly diagnosed in middle-aged individuals, with a slight male predominance and a common location in the anterior mandible [8]. GOCs are mostly asymptomatic until they cause swelling and cortical expansion. Radiologically, they present as well-defined unilocular or multilocular radiolucencies, sometimes with root resorption or maxillary sinus involvement [9]. Intraoral examination of the present case revealed a localized elevation due to mild expansion of the alveolar process in the right maxilla.

MEC is an uncommon salivary gland neoplasm that occurs predominantly in the major salivary glands and less commonly in the minor salivary glands, with the palate being the most frequent location. Clinically, palatal MEC presents as a firm, painless swelling [10], as observed in this case. Histopathologically, MECs are graded as low-, intermediate-, and high-grade tumors, with the current case exhibiting features consistent with a low-grade tumor.

For small, low-grade tumors without bone involvement, wide excision extending to the periosteum with 1-2 cm tumor-free lateral margins is considered adequate treatment [11]. The standard treatment for all grades of MEC is surgical resection. For MECs of minor salivary glands with low- to intermediate-grade, wide local excision with adequate tumor-free margins is the preferred treatment. High-grade tumors require more aggressive surgery, potentially accompanied by postoperative radiotherapy and chemotherapy [12].

For low- to intermediate-grade MECs originating in intraoral minor salivary glands, recurrence is uncommon (~10%), and patient survival rates are reported to be very high (95-100%) [10]. No evidence of metastasis to regional lymph nodes was detected in the CECT of the head and neck.

Management strategies differed according to the biological behavior of each lesion. Enucleation was chosen for the GOC, consistent with standard treatment for localized lesions without cortical perforation. Conversely, the LGMEC required wide local excision to ensure adequate surgical margins and reduce recurrence risk. The favorable postoperative outcome and lack of recurrence during follow-up highlight the importance of individualized, pathology-based surgical planning.

## Conclusions

This case emphasizes the critical importance of comprehensive clinical evaluation, detailed radiographic assessment, and meticulous histopathological examination in patients presenting with maxillary swellings. Lesions occurring in the maxillofacial region often exhibit overlapping clinical and radiographic features, making definitive diagnosis challenging. The rare simultaneous occurrence of a GOC and an LGMEC in the same anatomical region highlights the necessity of maintaining a broad differential diagnosis, particularly when the clinical presentation appears unusual or atypical. Such coexistence of distinct pathological entities may lead to diagnostic dilemmas and underscores the indispensable role of histopathological analysis in establishing an accurate diagnosis. Furthermore, this case demonstrates that accurate diagnosis followed by lesion-specific surgical management can result in successful disease control, favorable functional and aesthetic outcomes, and minimal postoperative morbidity. Therefore, a multidisciplinary approach involving careful clinical assessment, imaging, pathology, and tailored surgical intervention remains paramount in the management of complex maxillary lesions.
